# The Role of Natural Killer Cells and CD8^+^ T Cells in Hepatitis B Virus Infection

**DOI:** 10.3389/fimmu.2014.00258

**Published:** 2014-06-03

**Authors:** Anita Schuch, Alexander Hoh, Robert Thimme

**Affiliations:** ^1^Department of Medicine II, University Hospital of Freiburg, Freiburg, Germany; ^2^Faculty of Biology, University of Freiburg, Freiburg, Germany; ^3^Spemann Graduate School of Biology and Medicine, University of Freiburg, Freiburg, Germany

**Keywords:** natural killer cells, CD8^+^ T cells, hepatitis B virus, functional dichotomy, T cell failure

## Abstract

Hepatitis B virus (HBV) infection is one of the main causes of chronic liver diseases that may progress to liver cirrhosis and hepatocellular carcinoma. Host immune responses are important factors that determine whether HBV infection is cleared or persists. Natural killer (NK) cells represent the main effector population of the innate immune system and are abundant in the human liver. Recently, it has been demonstrated that NK cells not only exhibit antiviral functions but may also regulate adaptive immune responses by deletion of HBV-specific CD8^+^ T cells. It is well-established that HBV-specific CD8^+^ T cells contribute to virus elimination. However, the mechanisms contributing to CD8^+^ T cell failure in chronic HBV infection are not well-understood. In this review, we will summarize the current knowledge about NK cells and CD8^+^ T cells and illustrate their contribution to viral clearance and persistence in HBV infection. Moreover, novel immunological *in vitro* model systems and techniques to analyze HBV-specific CD8^+^ T cells, which are barely detectable using current multimer staining methods, will be discussed.

## Introduction

Hepatitis B virus (HBV) infection represents a major health care problem that affects around 350 million people worldwide, despite the availability of a prophylactic vaccine (1). The course of infection can be either acute or chronic, while persistence rate is considerably higher when HBV is acquired at birth or early infancy. Chronically infected patients are at risk of developing HBV-related diseases such as liver cirrhosis and hepatocellular carcinoma that account for 600,000 deaths annually (1). Although potent antiviral drugs such as nucleos(t)ide analogs and pegylated interferon-α (pegIFN-α) are available, treatment is rarely curative and patients often receive life-long therapy with the potential emergence of resistance and toxicity.

Natural killer (NK) cells, as part of the innate immune system, represent the first line of defense against viral infections. In addition to their antiviral effector functions, NK cells may also interact with and thereby negatively regulate HBV-specific CD8^+^ T cells (2). CD8^+^ T cells are thought to be the main effector cells since their experimental depletion delays the clearance of acute HBV infection in chimpanzees (3). Of note, persistent infection is characterized by impaired HBV-specific CD8^+^ T cell responses (4). The mechanisms responsible for this CD8^+^ T cell failure are less understood. Interestingly, different mechanisms that may lead to impaired and dysfunctional HBV-specific CD8^+^ T cell responses in chronically infected patients have been reported. Therefore, it is plausible that at least a part of immune-mediated liver damage is due to immune cells other than virus-specific T cells (5).

In the present review, we will focus on the role of NK cells and HBV-specific CD8^+^ T cells, which are thought to be responsible for both virus control and disease pathogenesis.

## NK Cells

Natural killer cells represent the main effector cell population involved in innate immune responses against intracellular pathogens and abnormal cells (6). They are enriched in the liver (7) and account for one-third of the intrahepatic lymphocytes compared to 5–15% in the peripheral blood (8).

Natural killer cells do not express recombination-dependent antigen-specific receptors, therefore it is assumed that stimulation of NK cells is antigen-independent (9). However, NK cells have several traits in common with CD8^+^ T cells (10, 11): they share a common bipotential progenitor and exert similar killing mechanisms. Additionally, a number of cell surface molecules, referred to as “NK receptors” are also expressed on activated CD8^+^ T cells. Furthermore, murine NK cells have been described to mediate long-lived hapten-specific recall responses leading to the assumption of a NK cell memory (12).

Natural killer cell activation is regulated by the interplay of several activating and inhibitory receptors (10, 13) and cytokines such as type I IFN, interleukin (IL)-2, IL-12, IL-15, and IL-18 (14, 15). The best characterized activating receptor on the surface of NK cells is NKG2D. This receptor recognizes molecules, that are expressed at low levels on most cells but are upregulated upon infection or stress, such as MICA, MICB, and RAET1 proteins (10, 16, 17). Furthermore, NKp46 (18), NKp44 (19), and NKp30 (20) are important receptors involved in target cell recognition and killing (21). Another important NK cell stimulatory receptor is CD16, also known as the Fc receptor FcγRIII, which triggers antibody-dependent cell cytotoxicity (22). Moreover, NK cells are regulated by inhibitory receptors, which are known to mainly engage major histocompatibility complex (MHC) class I molecules expressed on the surface of target cells (23). Some of these inhibitory markers belong to a distinct family of receptors termed killer cell immunoglobulin-like receptors (KIRs), which include both activating and inhibitory molecules (22). Additionally, the CD94/NKG2A heterodimeric receptor is often used as an inhibitory marker (24).

Depending on the distribution of activating versus inhibitory signals and the prevalent cytokine milieu NK cells display at least two effector functions: they are able to produce a variety of antiviral active and immunoregulatory cytokines such as IFN-γ, tumor necrosis factor (TNF), granulocyte–macrophage colony stimulating factor (GM-CSF), and IL-10 (25, 26) and they can directly kill target cells through the release of perforin and granzymes at immunological synapses (27). Since hepatocytes are considered to be relatively resistant to cytotoxicity of NK cells via the perforin/granzyme pathway, tumor-necrosis-factor-related-apoptosis-inducing ligand (TRAIL) is likely to play a major role in hepatocellular damage (28).

Based on their expression of CD56, two NK cell subpopulations can be defined: CD56^dim^ and CD56^bright^. CD56^dim^ NK cells represent the major circulating subset and are regarded as developmentally mature. Furthermore, this subset is thought to exert mainly cytotoxic effector functions (29), although it has been shown that CD56^dim^ NK cells are also able to produce large amounts of IFN-γ during the first hours after stimulation (30). By contrast, CD56^bright^ NK cells represent an earlier stage of maturation. They comprise the minority in the peripheral blood and are considered as the main cytokine producers (29, 31–33). However, viral infections may alter these proportions, leading to a relative enrichment of the CD56^bright^ population (34, 35).

Next to their antiviral function, NK cells have also been shown to regulate other immune cells thereby shaping both innate and adaptive immune responses. Indeed, various studies focused on the interaction between NK cells and innate immune cells such as monocyte-derived dendritic cells, plasmacytoid dendritic cells (pDCs), and macrophages. This crosstalk can modulate NK cell functions by direct cell-to-cell contact or the activity of soluble factors (36). For example, NK cell interaction with macrophages and DCs via CD40L/CD40 drives production of IL-12, which in turn not only induces NK cells to produce IFN-γ but also enhances NK cell cytotoxicity (37–39). Additionally, NK cell function can also be improved by type I IFN abundantly secreted by pDCs (40). Furthermore, NK cells can also interact with components of the adaptive immune system and may limit CD8^+^ T cell responses, as it has been shown in LCMV infection (41–43).

Below, we will summarize the current knowledge about of NK cells in acute and chronic HBV infection and discuss their role in regulating HBV-specific CD8^+^ T cell immunity.

## NK Cells in Acute HBV Infection

Viral replication usually results in the activation of an innate immune response that is characterized by the rapid production of type I IFN. These cytokines induce the expression of interferon-stimulated genes (ISGs), which in turn exert several intracellular antiviral mechanisms to limit viral spread, including the upregulation of MHC I molecules on the surface of infected cells (44). However, HBV seems not to induce any detectable intrahepatic expression of ISGs in chimpanzees during the first weeks of infection and therefore has been postulated to be a “stealth virus” that does not activate the innate immune system (45). This assumption has been challenged *in vitro* by the finding that HBV replication elicits a strong and specific innate antiviral response in HepaRG cells with an upregulation of IFN-β and other ISGs resulting in a non-cytopathic clearance of HBV DNA (46). Furthermore, a significant reduction in HBV DNA has been reported in acutely infected chimpanzees long before the peak of T cell infiltration and liver damage, suggesting a contribution of non-cytopathic antiviral mechanisms to viral clearance (47). The influx of NK cells that recognize infected cells in the absence of MHC I expression has been suggested to contribute in this setting. Moreover, the induction of IFN-γ and TNF in the liver of chimpanzees during the described non-cytopathic pre-T cell phase of viral clearance supports this hypothesis, because these effector cytokines are produced not only by CD8^+^ T cells but also by NK cells.

Since the incubation period of acute HBV infection is predominantly asymptomatic and therefore difficult to study, only limited and partially contradicting information about the role of NK cells during the early stages of infection is available in humans. One of the leading studies was performed during the preclinical phase in two subjects with acute HBV infection characterized by persistently normal alanine aminotransferase (ALT) levels (48). NK cells were promptly activated before peak viremia occurred, as indicated by the early increase of NK cells expressing the activation markers CD69 and NKG2D. According to this, the highest number of circulating NK cells was found at an early stage in the incubation period of patients with acute HBV infection (49).

However, an impaired NK cell function in patients with acute hepatitis B has also been reported. Indeed, Dunn et al. showed that NK cell activation in acutely HBV-infected patients is significantly inhibited compared to healthy subjects, especially during the time of peak viremia (50). High viral load was also associated with a reduction of rather non-cytolytic than cytolytic NK cell effector functions. In addition, type I IFN, IFN-λ1, and IL-15, essential activators of NK cells, were barely detectable in these patients, supporting the dogma of HBV being a stealth virus. However, IL-10 levels increased early in the course of infection and the highest concentration was found at the time of peak viremia when NK cell IFN-γ production was severely reduced. This suggests a role for IL-10 in the inhibition of NK cell antiviral responses. The authors confirmed *in vitro* that addition of exogenous IL-10 to activated NK cells induces significant suppression of NK cell-derived IFN-γ, while blocking of IL-10 restored NK cell effector function (50).

However, it has also been reported that NK cells exert higher cytolytic activity and IFN-γ production during acute HBV infection. This was concomitant with the elevated expression of activating receptors such as NKp46, and lower levels of inhibitory markers, e.g., NKG2A (34). Furthermore, NK cell activation, measured by the expression of CD69, CD38, and HLA-DR, was correlated positively with ALT levels and negatively with viral load, suggesting a close association of activated NK cells with liver necroinflammation and HBV clearance in acute HBV infection. In addition to the altered phenotype, the frequency and subset distribution was also modified in patients with acute hepatitis B, showing a significant enrichment of CD56^bright^ NK cells (34, 35).

The discrepancy between these different studies may arise from the fact that disease progression in the analyzed patients was either asymptomatic or symptomatic in concert with normal and elevated ALT levels, respectively. Overall, these results point to an important role of NK cells that are activated during acute HBV infection but might be functionally suppressed.

## NK Cells in Chronic HBV Infection

Studies regarding phenotype and function of NK cells during chronic HBV infection have revealed, in part, conflicting results. Several reports conclude that NK cells exhibit selective defects in their antiviral function. This functional dichotomy features a conserved or enhanced cytolytic activity (51, 52) and a diminished cytokine production (51, 53) that may contribute to viral persistence and implicate a role for NK cells in disease pathogenesis. The mechanisms leading to this functional impairment are still not fully understood but thought to be heterogeneous.

Hepatitis B virus infection may alter the activation status and receptor expression patterns on the surface of NK cells. Indeed, the expression of inhibitory receptors such as NKG2A is elevated while activating receptors, CD16 and NKp30, are downregulated (53, 54) and this correlates with serum HBV DNA load. Interestingly, antiviral therapy partially restores NK cell phenotype and functionality (53). However, these findings are controversial, since Bonorio et al. showed decreased levels of NKG2A-expressing NK cells in chronic HBV infection (55) and it was also reported, that HBV infection does not alter NKG2A expression on NK cells (35). In addition to classical NK cell receptors other co-inhibitory molecules involved in immune responses may impair NK cell function. Of note, T cell immunoglobulin- and mucin-domain-containing molecule-3 (Tim-3) has been shown to be upregulated on NK cells during HBV infection and *in vitro* blockade was able to enhance NK cell cytotoxicity (56).

An impaired NK cell activation and function may also arise from modified expression patterns of ligands for inhibitory and activating NK cell receptors. Indeed, it has been shown that the decreased expression of NKG2D ligands, MICA/B, on HBV-infected hepatocytes inhibits NK cell lysis (57).

Furthermore, the immunosuppressive cytokine environment in chronic HBV infection, created through high levels of IL-10, may inhibit the ability of NK cells to produce IFN-γ (58), as has already been shown in acutely infected patients (50). This defect persists in patients with chronic HBV infection receiving antiviral therapy, but can be reversed *in vitro* by specific blockade of IL-10 and transforming growth factor (TGF)-β (58).

In addition, the interaction with other immune cells may alter the reactivity of NK cells during persistent viral infection. For example, several studies have revealed that HBV interferes with pDCs, thereby modulating pDC-NK cell crosstalk *in vivo* and *in vitro* (59–61). Although circulating and intrahepatic pDCs from patients with chronic HBV infection showed a more activated phenotype, their ability to respond to toll-like receptor (TLR) 9 stimulation was significantly impaired (60). Moreover, patient-derived mature pDCs were poor activators of NK cell cytotoxic function due to their impaired IFN-α secretion and reduced OX40L expression. HBV seems not only to directly inhibit pDC maturation in a TLR9-dependent manner, but also to abrogate the supporting function of monocytes regarding IFN-α production by pDCs (59).

As mentioned above, NK cells may also exert regulatory functions (41–43). This is supported by a study in patients with chronic HBV infection, where *in vitro* depletion of NK cells increased HBV- but not CMV-specific CD8^+^ T cell responses (2). Elevated expression of TRAIL receptor 2 (TRAIL-R2) renders HBV-specific CD8^+^ T cells more susceptible to apoptosis by TRAIL-expressing NK cells. TRAIL-R2-expression patterns correlated with HBV DNA titer, thus the regulatory role of NK cells may be relevant during HBV flares. Longitudinal analysis of chronically HBV-infected patients already revealed a temporal correlation between ALT flares and TRAIL-expressing NK cells (62).

Taken together, these findings suggest that NK cells may exert a non-classical regulatory next to their classical antiviral function in HBV infection.

## CD8^+^ T Cells

CD8^+^ T cells are a major component of cellular adaptive immunity. They normally mediate protection against intracellular pathogens and tumor cells. In order to be properly activated, CD8^+^ T cells require at least two signals: first, the recognition of their cognate antigen presented by MHC I molecules on antigen-presenting cells (APCs). This is mediated by the interaction of the antigen-specific T cell receptor (TCR) with peptide-MHC I complexes. Second, additional co-stimulatory signals have to be provided by the same APC to prevent anergy. Furthermore, different cytokine milieus may influence this activation process (63). Upon antigen-recognition, naïve CD8^+^ T cells undergo clonal expansion and differentiate into cytotoxic effector and memory T cells (64). According to their differentiation status, they are characterized by distinct expression patterns of surface markers such as CD45RA, CD27, CD28, and CCR7 (65). Similar to NK cells, the effector functions of CD8^+^ T cells comprise several mechanisms such as the secretion of cytokines (IFN-γ and TNF), the release of cytotoxic mediators (perforin/granzyme), and receptor-mediated induction of apoptosis (e.g., through TRAIL) (63). In the following, we will elaborate the current knowledge about phenotype and function of CD8^+^ T cells in the context of acute and chronic HBV infection.

## CD8^+^ T Cells in Acute HBV Infection

Hepatitis B virus-specific CD8^+^ T cells play a major role in controlling and resolving HBV infection. Indeed, strong HBV-specific CD8^+^ T cell responses have been shown to correlate with viral clearance during acute infection (66). The antiviral role of CD8^+^ T cells has been further confirmed by depletion studies in experimentally infected chimpanzees, where in the absence of CD8^+^ T cells virus titer remained at high levels (3). Importantly, the re-appearance of CD8^+^ T cells in the circulation coincided with a decrease in viremia and the onset of liver disease. However, it has been shown that virus-specific CD8^+^ T cells are functionally impaired during the acute phase until infection is resolved (50, 66, 67). Furthermore, the mechanisms of CD8^+^ T cell-mediated antiviral control are still debated.

Indeed, studies in the transgenic mouse model revealed that HBV-specific CD8^+^ T cells are able to abolish viral replication in the liver while killing only a small fraction of hepatocytes (68). This was mediated by inflammatory cytokines such as IFN-γ and TNF. The contribution of non-cytolytic effector mechanisms has been further supported by findings in a cell culture model, where virus-specific CD8^+^ T cells were able to inhibit HBV replication in HepG2 2.2.15 cells with only minimal cell lysis (69). Of note, the authors could show that particularly IFN-γ and TNF are responsible for HBV inactivation in target cells, since blocking of these two cytokines abrogated the non-cytolytic inhibition of virus replication. Furthermore, in acutely infected chimpanzees most of HBV DNA elimination has been shown to occur before the peak of T cell infiltration and liver injury, also suggesting non-cytolytic effector mechanisms like the secretion of IFN-γ and TNF (47). Still, the contribution of cytolytic effects is supported by several studies (3, 70).

The development of cell culture models permits the investigation of the relative importance of cytolytic versus non-cytolytic effector functions and their impact on the suppression of HBV replication (71). Various cell lines transfected with the HBV genome have been established, e.g., HepG2.117 (72), HepG2.2.15 (73), or HepAD38 (74). Although the secreted virions are infectious, these cell lines are still quite artificial, since none of them is susceptible for HBV infection. Until the recent discovery of human sodium taurocholate cotransporting polypeptide (hNTCP) as HBV entry receptor (75, 76) *in vitro* infection could only be conducted in primary human hepatocytes, primary tupaia hepatocytes, and in differentiated HepaRG cells. The latter was the first hepatoma cell line being susceptible to HBV and supporting the full viral replication cycle after DMSO-dependent differentiation (77). However, cultures of differentiated HepaRG cells represent a mixture of biliary-like epithelial cells and hepatocyte-like cells and only the latter subset was shown to be susceptible for HBV infection with an infection efficacy below 20% (78). Hepatoma cell lines such as HuH7 and HepG2 cells lack hNTCP expression and are therefore non-permissive to HBV infection. Of note, infection rates with up to 70% can be achieved when HepG2 cells are transduced with hNTCP (76). These novel cell lines may be useful not only to study the molecular virology of HBV but also to improve our understanding of the relative contribution of cytolytic and non-cytolytic effector functions to viral clearance.

## CD8^+^ T Cells in Chronic HBV Infection

In chronically HBV-infected individuals, virus-specific CD8^+^ T cell responses are rarely detectable (5, 79–82). The profiles of HBV-specific CD8^+^ T cell responses depend on the stage of disease and are highly influenced by the level of HBV replication. Indeed, circulating multispecific HBV-specific CD8^+^ T cell responses are predominantly detectable *ex vivo* in patients with low viral load. In individuals with a high level of HBV replication (>10^7^ copies/ml) virus-specific CD8^+^ T cells were occasionally detectable only after *in vitro* expansion (81). However, the mechanisms responsible for the lack of functional CD8^+^ T cell responses are not completely understood. It might be that virus-specific CD8^+^ T cells are deleted. Indeed, the elevated intracellular expression of the pro-apoptotic protein Bcl2-interacting mediator (Bim) in HBV-specific CD8^+^ T cells of chronically infected patients supports this hypothesis (83).

Furthermore, HBV-specific CD8^+^ T cells may not or only insufficiently be primed by APCs and consequently may not expand upon antigen-encounter. Of note, several studies suggest a dysfunction of DCs in chronically HBV-infected patients, including reduced expression of co-stimulatory molecules, impaired cytokine secretion, and lower allostimulatory capacity compared to healthy subjects (84–87). According to this, HBV-specific CD8^+^ T cells would be expected to display a naïve phenotype, characterized by high expression levels of CD45RA, CD27, CD28, and CCR7 (65).

In addition, it is possible that they are not traceable since the frequencies of virus-specific CD8^+^ T cell responses are below the detection limit of conventional quantitative assays, as it has been shown in chronic HCV infection (Schmidt, unpublished data). New techniques for enumerating epitope-specific T cells from human peripheral blood based on the combination of tetramer staining, magnetic-bead enrichment, and multiparametric flow cytometry (88) may address at least the latter two possibilities.

Virus-specific CD8^+^ T cells isolated from the peripheral blood of chronically HBV-infected patients are functionally impaired and seem to have lost most of their ability to proliferate and to produce cytokines, like IFN-γ (82). The reported CD8^+^ T cell failure has been attributed to high levels of persisting viral antigens. Despite the constant presentation of viral peptides on MHC molecules, circulating sub-viral particles, comprised of soluble HBV surface antigen (HBsAg) along with HBeAg may drive chronic T cell stimulation. Particularly, the latter has been implicated in altering the reactivity of virus-specific CD8^+^ T cells (89). Moreover, accessory HBsAg seroconversion has been reported to induce a more potent restoration of CD8^+^ T cell responses than HBV viral load reduction alone (90).

CD8^+^ T cell dysfunction in chronic HBV infection follows a well-established pattern with elevated expression of inhibitory molecules such as programmed death-1 (PD-1) (82, 91), cytotoxic T lymphocyte antigen 4 (CTLA-4) (92), Tim-3 (93, 94), and 2B4 (CD244) (95) on T cells. Furthermore, the expression of corresponding ligands such as PD ligand (PD-L)1 has been shown to be increased on hepatocytes (96). According to this, highly viremic HBV-infected patients show a more severely impaired CD8^+^ T cell phenotype and T cell dysfunction is more profound in the liver than in the blood (91, 97). Blockade of these inhibitory pathways may at least partially restore HBV-specific CD8^+^ T cell functionality, as it has been shown *in vitro* (82, 91, 92, 94, 95). The potential relevance of blocking the PD-1 pathway was demonstrated in the HBV mouse model where HBV-transgenic mice were treated with blocking antibodies for PD-L1 prior to the adoptive transfer of HBV-specific cytotoxic T cells (98). This treatment resulted in an increased number of IFN-γ-producing CD8^+^ T cells in the liver and in a delayed suppression of these CD8^+^ T cells. Furthermore, a recent study could show that *in vivo* blockade of the PD-1/PD-L1 pathway, together with entecavir treatment and DNA vaccination, enhances virus-specific CD8^+^ T cell responses in the woodchuck model, leading to sustained immunological control of viral infection (99).

The combined modulation of these inhibitory pathways along with the activation of co-stimulatory pathways might be beneficial (92, 95, 100, 101). However, a more detailed insight into the relative contribution of individual inhibitory pathways to HBV-specific CD8^+^ T cell dysfunction and concomitant the impact of inhibitory receptor blockade on restoration of CD8^+^ T cell responses is necessary.

Moreover, the lack of CD4^+^ T cell help contributes to defective CD8^+^ T cell function (102). Increased regulatory T cell numbers (103–105), together with immunosuppressive cytokines such as IL-10 and TGF-β (50, 58, 106) impair virus-specific CD8^+^ T cell responses. It has also been reported that increased intrahepatic arginase levels (67, 107) and hence the lack of arginine lead to a functional silencing of CD8^+^ T cells due to the downregulation of the CD3ζ-chain (108).

Collectively, these studies demonstrate that several mechanisms may contribute to the diminished frequency and function of virus-specific CD8^+^ T cells in the chronic phase of HBV infection and that combined modulation of different pathways may lead to a restoration of HBV-specific T cell responses.

## Conclusion

**Figure 1:**
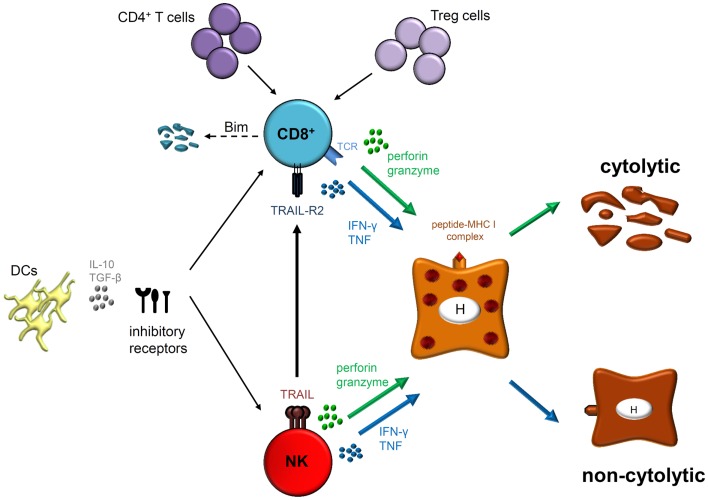
**Natural killer cells as well as HBV-specific CD8^+^ T cells exhibit their antiviral functions by cytolytic (perforin and granzyme) and/or non-cytolytic (IFN-γ and TNF) mechanisms**. However, CD8^+^ T cells express antigen-specific T cell receptors (TCRs) that interact with peptide-MHC I complexes on infected hepatocytes (H) whereas NK cell activation is thought to be antigen-independent. Effector functions and phenotype of both cell types are modulated during acute and chronic HBV infection. Indeed, different mechanisms play a role in regulating both effector populations, such as DCs, immunoregulatory cytokines (IL-10 and/or TGF-β) and expression of several inhibitory receptors. Furthermore, lack of CD4^+^ T cell help and interaction with regulatory T (Treg) cells may lead to CD8^+^ T cell dysfunction in chronically HBV-infected patients resulting in Bim-mediated apoptosis. Importantly, NK cells are also able to inhibit antiviral T cell responses by deleting HBV-specific CD8^+^ T cells in a TRAIL-dependent manner.

CD8^+^ T cell responses play an important role in HBV infection and contribute not only to viral clearance but also to liver injury. In the setting of chronic infection, several mechanisms of T cell dysfunction including expression of inhibitory molecules and pro-apoptotic proteins, as well as suppressive cell subsets and cytokines favor viral persistence (Figure 1). In addition, even antivirally active NK cells, which exert a unique influence in the early defense against HBV are supposed to control CD8^+^ T cells, particularly during hepatic flares.

Cell culture models taking advantage of hepatoma cell lines that are transduced with the recently identified HBV entry receptor, hNTCP, may allow novel insights into HBV immunobiology and pathogenesis, revealing the relative contribution of cytolytic and non-cytolytic mechanisms to viral clearance. Furthermore, enrichment techniques could uncover whether HBV-specific CD8^+^ T cells are actually deleted in chronically HBV-infected patients and could also elucidate the phenotype of the detectable virus-specific CD8^+^ T cells. A better understanding of the mechanisms leading to viral persistence may result in new therapeutic treatment strategies that aim to remedy the T cell defects described, thereby augmenting functional responses and decreasing antigen-unspecific liver damage.

## Conflict of Interest Statement

The authors declare that the research was conducted in the absence of any commercial or financial relationships that could be construed as a potential conflict of interest.
